# Diagnostic performance of standard breast MR imaging compared to dedicated axillary MR imaging in the evaluation of axillary lymph node

**DOI:** 10.1186/s12880-020-00449-4

**Published:** 2020-05-01

**Authors:** Su Min Ha, Eun Young Chae, Joo Hee Cha, Hee Jung Shin, Woo Jung Choi, Hak Hee Kim

**Affiliations:** 1 Department of Radiology, Research Institute of Radiology, Asan Medical Center, University of Ulsan College of Medicine, 88 Olympic-ro 43 gil, Songpa-gu, Seoul, 05505 South Korea; 2 Department of Radiology, Seoul National University Hospital, 28 Yongon-dong, Chongno-gu, Seoul, 110-744 South Korea

**Keywords:** Axilla, Breast cancer, Magnetic resonance imaging

## Abstract

**Background:**

Breast magnetic resonance (MR) imaging does not usually assess axillary lymph nodes -using dedicated axillary sequence. The additional utility of dedicated axillary sequence is poorly understood. We evaluated the diagnostic performance of dedicated axillary imaging sequence for evaluation of axillary lymph node.

**Methods:**

In this retrospective study from January 2018 to March 2018, 750 consecutive women underwent breast MR imaging. 263 patients were excluded, due to neoadjuvant chemotherapy (*n* = 235), incomplete histopathological information (*n* = 14) and follow-up loss (n = 14), 487 women were included. Two radiologists scored lymph node on confidence level scale from 0 (definitely benign) to 4 (definitely malignant), −using standard MR and dedicated axillary imaging sequences. Diagnostic performance parameters were compared and calculated correlation coefficient of quantitative features (largest dimension, cortical thickness, and the ratio of cortical thickness to largest dimension of lymph node).

**Results:**

68 (14.0%) were node-positive and 419 (86.0%) were node-negative. The sensitivity, specificity, positive, negative predictive values and accuracy were respectively, 66.2, 93.3, 61.6, 94.4, and 89.5% for dedicated axillary sequence and 64.7, 94.0, 63.8, 94.3, 89.9% for standard MR sequence The dedicated axillary and standard sequences s did not exhibit significant differences in detection of positive lymph nodes (AUC, 0.794 for standard and 0.798 for dedicated axillary sequence, *P* = 0.825). The cortical thickness appeared to be the most discriminative quantitative measurement using both axillary (AUC, 0.846) and standard sequences (AUC, 0.823), with high correlation coefficient (0.947).

**Conclusion:**

Evaluation of axillary nodal status using standard breast MR imaging is comparable to dedicated axillary MR imaging.

## Background

The presence of axillary lymph nodes metastases in breast cancer patients helps determine surgical and postsurgical care and is recognized as one of the most important prognostic factors for overall survival [1]. However, recent studies such as ACOSOG Z0011, AATRM 048/13/2000 and IBCSG 23–01 trials have reported that completion of axillary lymph node dissection (ALND) in women with a limited number of axillary nodal disease (i.e, 1–3 positive nodes) cannot improve long term prognosis [2–4]. Rather, it is increasingly important to exclude advanced axillary node metastases (i.e, 3 or more positive nodes) than detecting clinically node positive disease. If there is a noninvasive imaging modality that can accurately determine node negative disease, omitting ALND and even sentinel lymph node biopsy (SLNB) could be considered, resulting in a significant reduction of morbidity.

Breast magnetic resonance (MR) imaging is a non-invasive imaging modality for axillary evaluation to identify node positive and negative disease in patients with breast cancer [5]. However, the use of MR imaging for the evaluation of axillary lymph nodes is controversial. Previous studies assessed lymph nodes seen at preoperative breast MR imaging instead of using dedicated high-spatial resolution axillary MR imaging sequences [6–9]. But others described that bilateral assessment of axillary lymph nodes and even the supraclavicular area was limited when using only dedicated breast coils [10–12]. The use of dedicated axillary MR imaging sequence has been shown to improve the accuracy of MR imaging in nodal evaluation [10, 13–15]. Although a dedicated examination of the axilla can be more accurate, it requires more scanning time [13, 16]. Indeed, there have been many various attempts to evaluate axillary lymph nodes. Baltzer et al. [14] insisted that technically axillary staging is feasible with conventional breast MR using a dedicated whole-body scanner. Another study also investigated gadofosveset enhanced MR imaging of axillary lymph nodes in breast cancer patients [15].

With increasing incidence of screening for breast cancer, more simplified and less time consuming examinations are demanded, which developed abbreviated MR protocol. The abbreviated MR has been considered to reduce scan time under ten minutes with including only pre-and immediate post contrast sequences with high diagnostic accuracy [17]. Although there has been several studies comparing the diagnostic performance of abbreviated MR protocols in breast cancer itself, there has been no previous study justifying for omission of dedicated axillary sequence. Thus, comparison of standard MR and dedicated axillary imaging sequences for axillary assessment in both screening and diagnostic setting is required. The purpose of our study was to evaluate the diagnostic performance of standard MR imaging compared to dedicated axillary MR imaging for the evaluation of axillary lymph node in patients who performed breast MR imaging for various reasons.

## Methods

### Patients

The institutional review board approved this retrospective study, and the requirement for written informed consent was waived. From January 2018 and March 2018, we identified a total of 750 consecutive patients who performed breast MR imaging, using a computer database at our institution. We excluded 263 patients who were treated with neoadjuvant chemotherapy (*n* = 235), had incomplete histopathological information (*n* = 14) and follow-up loss (n = 14). Finally, a total of 487 patients (age range, 23–90 years; mean age, 50.1 years) were included in this study.

### Breast MR technique

MR examinations were performed in the prone position using either a 1.5-T or 3-T scanner (Magnetom Avanto (*n* = 135) or Skyra (*n* = 301), Siemens Medical Solutions, Erlangen, Germany; Ingenia (*n* = 51), Philips Medical Systems, Best, The Netherlands) with a dedicated 18-channel phased-array breast coil (Supplementary Table 1). Standard imaging sequences included a T2-weighted sequence and a dynamic contrast material-enhanced fat-suppressed axial 3-dimensional T1-weighted sequence that included one precontrast-enhanced and 5 contrast-enhanced sequences. For axial T2-weighted imaging, a fast spin-echo sequence with fat suppression was used ([repetition time/echo time (TR/TE), 6700/74 msec; matrix size, 448 × 448; field of view (FOV) 300 × 300 mm; and slice thickness, 1.5 mm] for 1.5-T and [TR/TE, 1100/131 msec; matrix size, 256 × 416; FOV, 341 × 210 mm^2^; and slice thickness, 1.5 mm] for 3-T system). The dynamic contrast material-enhanced MR images were acquired with fast low-angle shot volume interpolated breath-hold examination (FLASH VIBE) pulse sequences ([TR/TE, 5.2/2.4 msec; matrix size, 384 × 384; slice thickness, 0.9 mm] for 1.5-T and [TR/TE, 5.6/2.5 msec; matrix size, 360 × 360; slice thickness, 0.9 mm] for 3-T scanner.

For further evaluation of nodal status, bilateral dedicated axial axillary imaging sequences were acquired using a body coil. A T1-weighted, contrast material-enhanced), fat-saturated, spoiled, volumetric-interpolated gradient-echo sequence was performed after a dynamic breast sequence. Imaging parameters were as follows: TR/TE, 5.6/2.6 ms; flip angle, 10°; matrix size, 298 × 352, slice thickness, 1.5 mm for 1.5-T scanner, TR/TE, 4.1/1.3 ms; flip angle, 12°; matrix size, 340 × 380; slice thickness, 1.0 mm; 160 slices for 3-T scanner. The field of view was optimized to involve the bilateral axillae (levels I–III), supraclavicular area, and inferior neck (levels IV–VII).

Contrast medium (0.1 mmol/kg; Dotarem, Guerbet, Aulnay-sous-Bois, France or 0.2 ml/kg; Uniray, Dongkook, Seoul, Korea) was injected at a flow rate of 1 or 2 ml/s followed by a 20-ml saline flush, using an MR-compatible power injector (Spectris; Medrad, Pittsburgh, PA).

### Image analysis

All patients were reviewed by two dedicated breast radiologists (S.M.H., and E.Y.C., with 7 and 9 years of clinical experience in breast imaging) in consensus. Image interpretation was performed in two reading sessions: (1) standard MR imaging and (2) dedicated axillary MR imaging sequences. We analyzed axillary lymph nodes first using standard MR imaging –sequence and then -using dedicated axillary MR sequence. The minimum interval between the reading sessions was 1 month to reduce the possible bias caused by the likelihood of readers remembering what they had previously read. The readers were blinded to the result of other reading session, clinical and histopathologic information.

The axillary lymph nodes were evaluated using standard MR imaging sequence consisting of axial T2-weighted and T1-weighted images obtained with fat saturation. If lymph node was suspected of metastasis [10, 11, 13, 18], the most suspicious node was selected and recorded for analysis. A lymph node was considered suspicious if it had one or more of the following features: (1) round or macrolobulated shape, which was determined when a node larger than 4 mm was not visible as an oval structure on two contiguous images; (2) loss of fatty hilum, with fat signal intensity not seen in the node; (3) uneven cortex, when a focal increased cortical thickness was noted but not placed in the center of the node but placed on one side; or (4) lobulated margin, when the node had an irregular outer contour. Using both MR imaging sequences, a confidence level scale of 0 (definitely benign) to 4 (definitely malignant) was recorded for each patient, by using the criteria as described by Baltzer et al. [14]. In addition, the largest dimension (LD) and the cortical thickness (CT) of the lymph node were measured and the ratio between cortical thickness and largest dimension of lymph node was calculated. When there was no suspicious lymph node in the axilla, the largest benign looking lymph node was selected for analysis. Dedicated axillary imaging sequence consisting of axial, fat saturated T1-weighted image was also evaluated in the same manner.

### Clinicohistopathologic analysis

The reference standard for axillary nodal status was a combination of pathological results (*n* = 340) and clinical follow-up results (*n* = 147) at least one year. The pathological data were reviewed from samples obtained by SLNB (*n* = 281), ALND (*n* = 38), core needle biopsy (*n* = 6) or fine needle aspiration (*n* = 15). Lymph node status was recorded as benign, isolated tumor cells (size≤0.2 mm), micrometastases (> 0.2 mm and ≤ 2.0 mm), or macrometastases (> 2.0 mm). For the purposes of this study, isolated tumor cells and micrometastases were considered as negative. In addition, the clinical indications for breast MR imaging was reviewed for each patient.

### Statistical analysis

Diagnostic performance parameters, including sensitivity, specificity, positive predictive value (PPV) and negative predictive value (NPV), were analyzed for standard MR imaging and dedicated axillary imaging sequences, using the confidence level scale. The confidence level scale for axillary nodal status was dichotomized, with lymph nodes scored 2 or lower considered as negative and those scored 3 and higher as positive test result. The diagnostic ability to identify patients with positive lymph nodes was also assessed based on the area under the receiver operating characteristic curve (AUC), the summary measure of the accuracy with AUC of 0.5, no ability to diagnose, 0.7–0.8 acceptable, 0.8–0.9, excellent and > 0.9 as outstanding performance. The diagnostic performance of both sequences was compared using the Exact binomial or Generalized score or McNemar’s test and AUC was compared using the DeLong test.

A Mann-Whiney U test or Student’s t-test was used to compare the quantitative measurements recorded (largest dimension, cortical thickness, and the ratio of cortical thickness to largest dimension) between positive and negative axillary lymph nodes. A correlation coefficient was calculated to evaluate the agreement of quantitative features (largest dimension, cortical thickness and the ratio of cortical thickness to largest dimension) between standard MR imaging and dedicated axillary MR imaging sequences. A *P* value of less than 0.05 was considered statistically significant. All statistical analyses were performed using SPSS software (version 23.0, Statistical Package for the Social Sciences, Chicago, IL).

## Result

### Patient characteristics

Of the 487 women who underwent breast MR imaging, 68 (14.0%, 68/487) were node-positive and 419 (86.0%, 419/487) were node-negative. The clinical indications for breast MR imaging included preoperative evaluation for known breast cancers in 354 patients, surveillance for women with a personal history of breast cancer in 106 patients, silicone implants or free injections in 14 patients, screening for high risk group with genetic mutation in 4 patients, or others in 9 patients. Among 354 patients with known breast cancers, the mean tumor size was 20.5 ± 16.6 mm (range, 1–103 mm). The tumors were predominantly invasive ductal carcinoma (222/354; 62.7%).

### Diagnostic performance of standard and axillary MR imaging for axillary lymph nodes

**Fig. 1:**
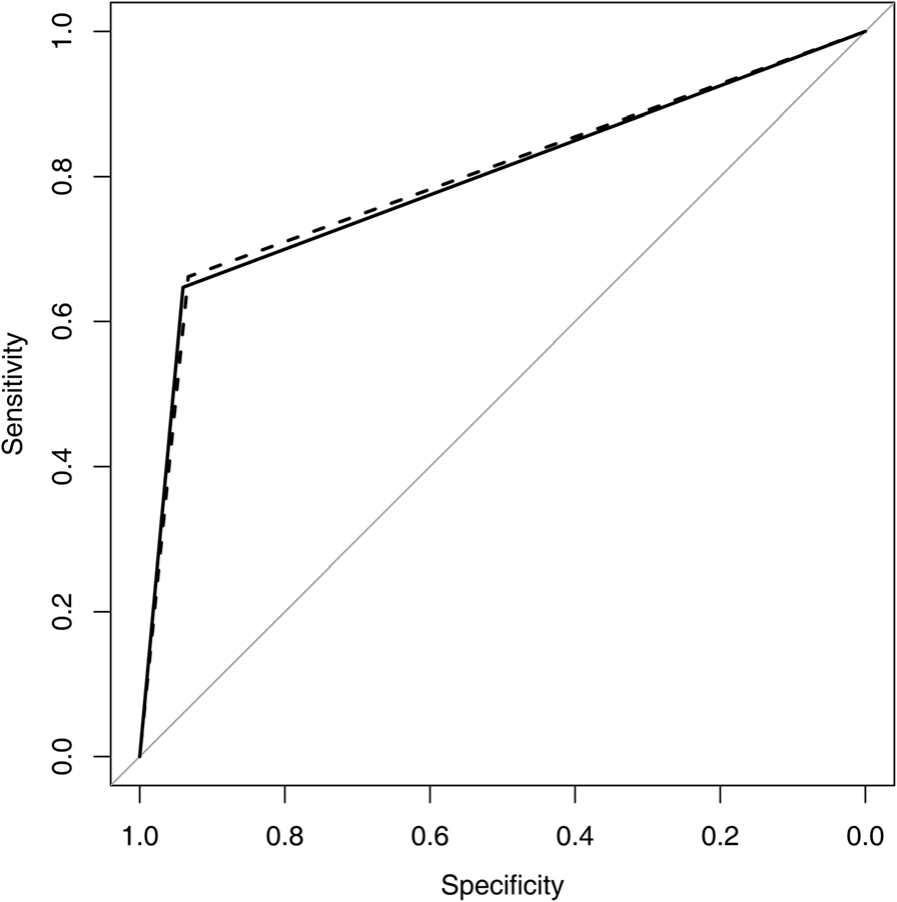
Receiver operating characteristic curves show qualitative assessment of standard (solid line) and dedicated axillary (dashed line) MR imaging sequence

Table 1 summarizes the diagnostic performance parameters of standard MR and dedicated axillary sequences for the evaluation of axillary nodal status. The standard MR sequence had the following diagnostic performance: sensitivity of 64.7%, specificity of 94.0%, PPV of 63.8%, NPV of 94.3% and accuracy of 89.9%. The dedicated axillary sequence showed comparable diagnostic performance: sensitivity of 66.2%, specificity of 93.3%, PPV of 61.6%, NPV of 94.4% and accuracy of 89.5%. There were no statistically significant differences between two sequences in comparison of sensitivity, specificity, PPV, NPV and accuracy (*P* > 0.05). The average AUCs were 0.794 for standard MR (95% confidence interval (CI), 0.735–0.852) and 0.798 for dedicated axillary sequence (95% CI, 0.740–0.855). The difference in AUC between two sequences was not statistically significant (*P* = 0.825) (Fig. 1).

**Table 1:** Performance Measures of Standard and Axillary MR Imaging Sequence for differentiation of positive and negative axillary lymph nodes

Parameters	Standard MR	Axillary MR	*P* value
Sensitivity	64.7 (52.2–75.9)	66.2 (53.7–77.2)	> 0.999
Specificity	94.0 (91.3–96.1)	93.3 (90.5–95.5)	0.581
Positive predictive value	63.8 (51.3–75.0)	61.6 (49.5–72.8)	0.531
Negative predictive value	94.3 (91.6–96.3)	94.4 (91.8–96.4)	0.715
Accuracy	89.9 (86.9–92.5)	89.5 (86.5–92.1)	0.814
Area under the curve	0.794 (0.735–0.852)	0.798 (0.740–0.855)	0.825

Note-Numbers in parenthesis are 95% confidence interval

### Quantitative measurement of standard and axillary MR imaging

Quantitative measurements, including the largest dimension (LD) and the cortical thickness (CT) of the lymph node and the ratio between CT and LD of lymph node, are summarized in Table 2. Using standard MR sequence, LD, CT and CT/LD ratio were found to be significantly higher in the node-positive group than in the node-negative group (all variables: *P* <  0.001). Using dedicated axillary sequence, these three quantitative measurements were also found to be significantly higher in the node-positive group than in the node-negative group (all variables: *P* <  0.001).

**Table 2:** Quantitative Measurements using Standard and Axillary MR Imaging Sequence

Parameter	Mean	Standard Deviation	Range	*P* value
**Standard MR**				
LD (mm)				< 0.001
Node Positive	15.90	9.60	5–46	
Node Negative	9.93	3.31	4–23	
CT (mm)				< 0.001
Node Positive	8.60	6.96	1–32	
Node Negative	3.26	1.30	1–9	
CT/LD ratio				< 0.001
Node Positive	0.52	0.20	0.07–1	
Node Negative	0.35	0.14	0.06–0.8	
**Axillary MR**				
LD (mm)				< 0.001
Node Positive	16.04	9.25	6–45	
Node Negative	10.18	3.14	5–22	
CT (mm)				< 0.001
Node Positive	9.13	7.10	2–35	
Node Negative	3.32	1.28	1–9	
CT/LD ratio				< 0.001
Node Positive	0.55	0.20	0.13–1	
Node Negative	0.35	0.14	0.06–0.78	

Note-*LD* largest dimension, *CT* cortical thickness

**Fig. 2:**
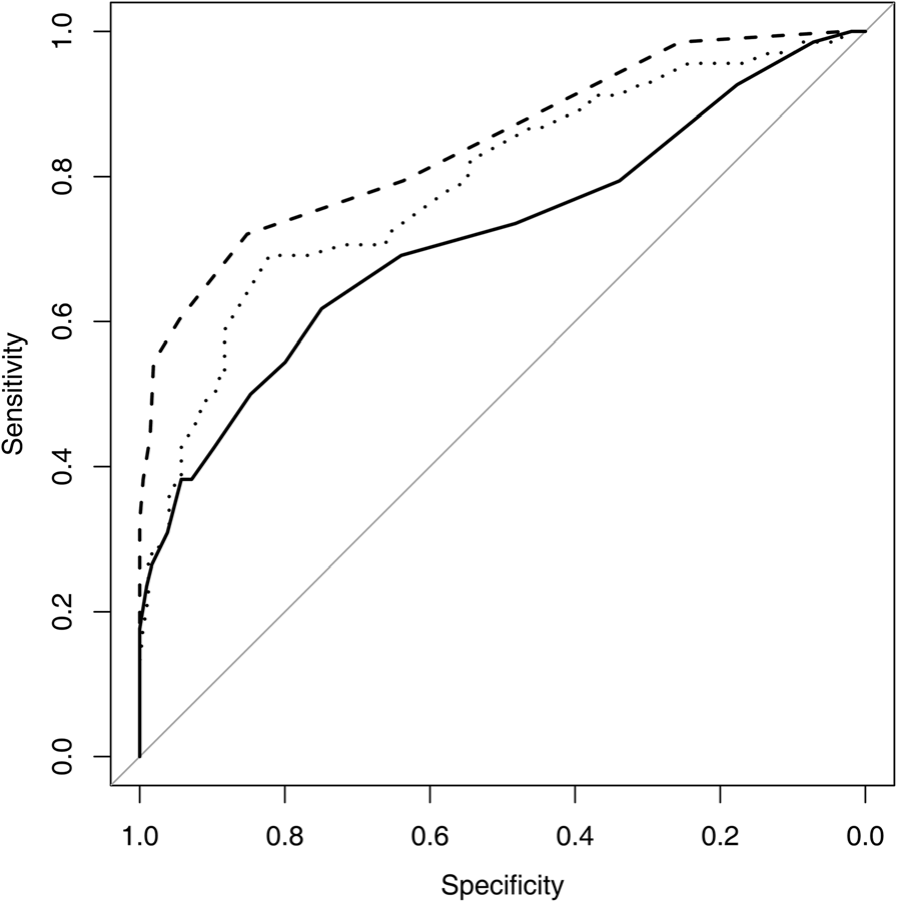
Receiver operating characteristic curves of axillary MR imaging sequence of largest dimension (solid line), cortical thickness (dashed line), and the ratio of cortical thickness to largest dimension (thin dots)

**Fig. 3:**
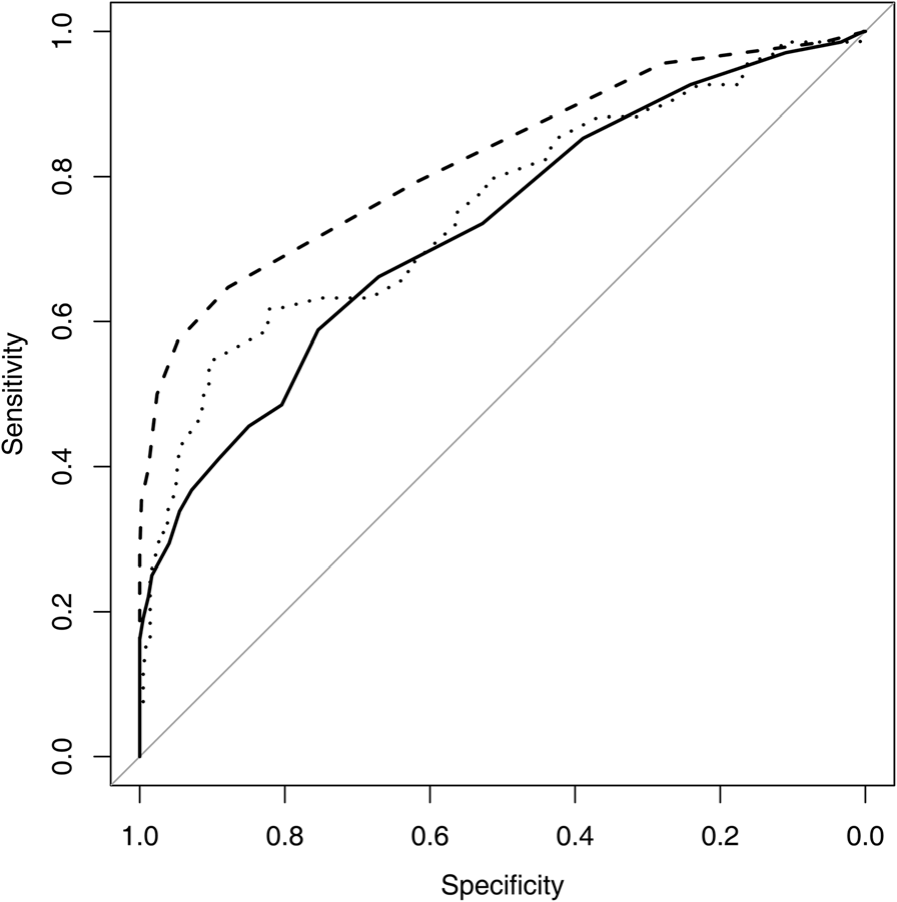
Receiver operating characteristic curves of standard MR imaging sequence of largest dimension (solid line), cortical thickness (dashed line), and the ratio of cortical thickness to largest dimension (thin dots)

Cortical thickness (CT) proved to be the most discriminative quantitative measurement to predict axillary lymph node metastasis (Table 3). The AUCs for CT were highest for both sequences, at 0.823 for the standard MR and 0.846 for the dedicated axillary. The AUCs for LD and CT/LD ratio were similar for both standard MR (0.727 vs. 0.754, *P* = 0.598) and dedicated axillary sequences (0.717 vs. 0.791, *P* = 0.155) (Fig. 2 and Fig. 3).

**Table 3:** Diagnostic Performance of largest dimension, cortical thickness and the ratio of cortical thickness to largest dimension using Standard MR and Axillary MR Imaging Sequence

Parameter	AUC	*P* value
Standard MR		
LD	0.727 (0.658–0.797)	0.007 ^a^
CT	0.823 (0.762–0.885)	0.005 ^b^
CT/LD ratio	0.754 (0.685–0.824)	0.598 ^c^
Axillary MR		
LD	0.717 (0.641–0.792)	< 0.001^a^
CT	0.846 (0.790–0.902)	0.016 ^b^
CT/LD ratio	0.791 (0.727–0.855)	0.155 ^c^

Note-Numbers in parenthesis are 95% confidence interval

*LD* Largest dimension, *CT* Cortical thickness

^a^ LD vs. CT, ^b^ CT vs. CT/LD ratio, ^c^ CT/LD ratio vs. LD

**Fig. 4:**
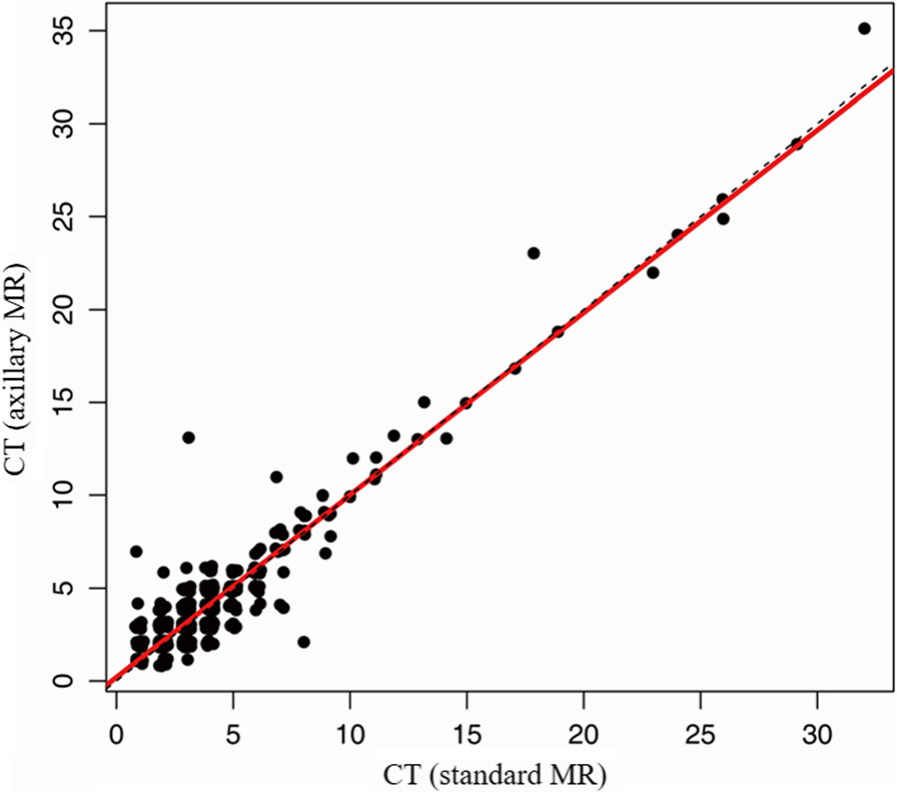
Correlation of Cortical thickness using Standard and Axillary MR imaging sequences

**Fig. 5:**
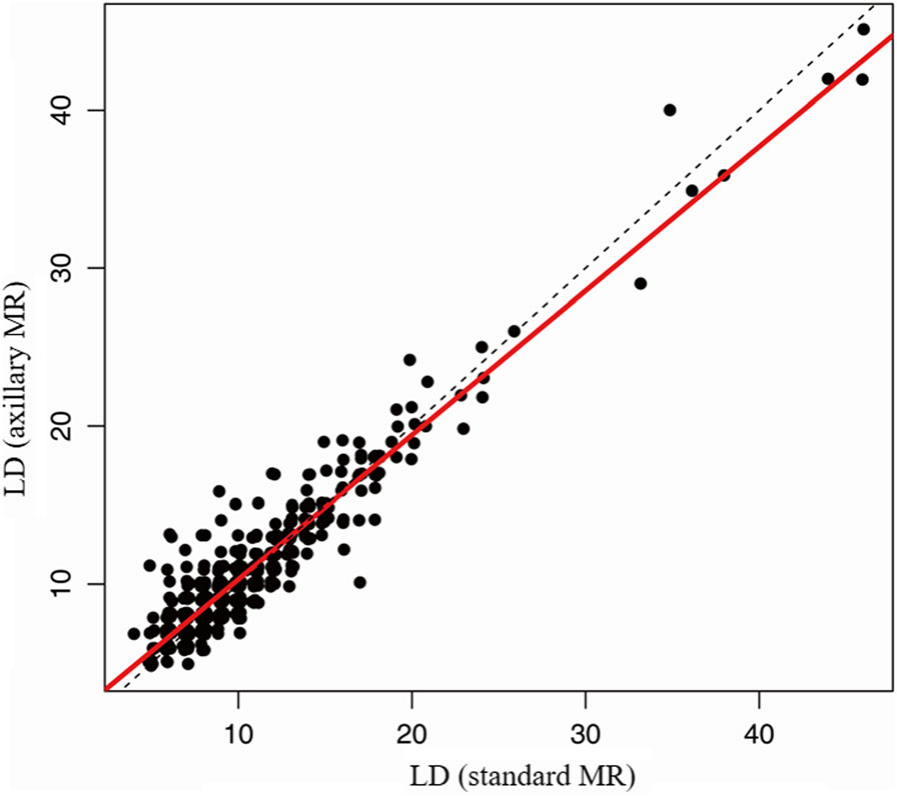
Correlation of Largest dimension using Standard and Axillary MR imaging sequences

**Fig. 6:**
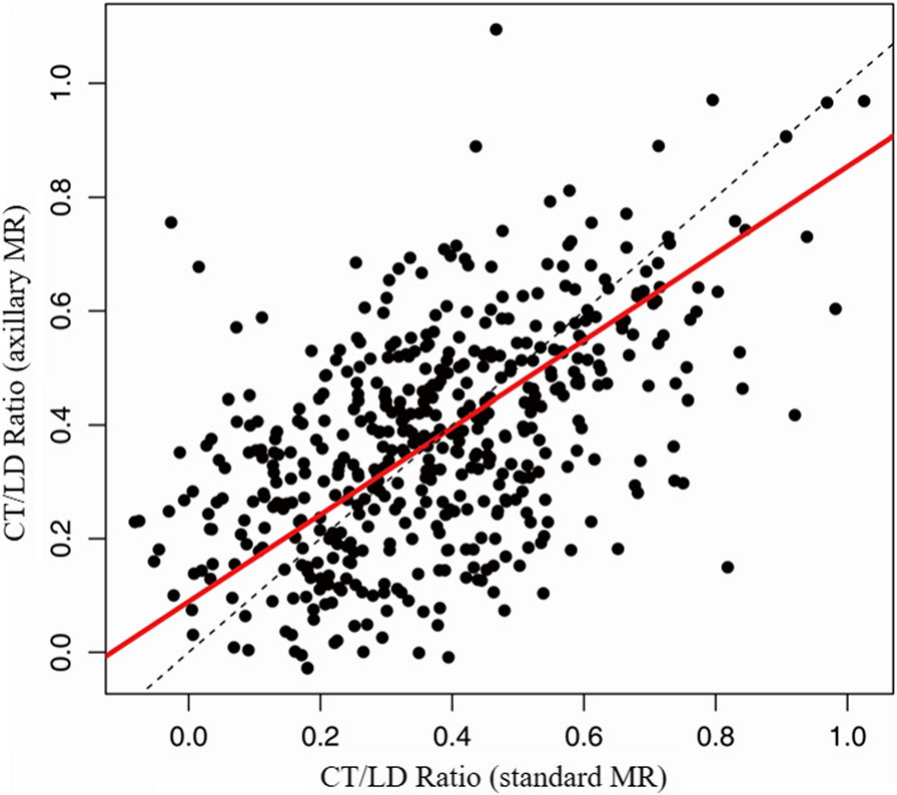
Correlation of Ratio of Cortical thickness to Largest Dimension using Standard and Axillary MR imaging sequences

The intra-class correlation coefficients of LD, CT and CT/LD ratio between two sequences were 0.949 (95% CI, 0.940–0.957), 0.947 (95% CI, 0.937–0.956) and 0.747 (95% CI, 0.705–0.784), respectively (Fig. 4, 5 and 6). All are > 0.70, which indicates strong agreement.

## Discussion

Our study demonstrated that there was no significant differences between standard MR and dedicated axillary MR sequences in the evaluation of axillary lymph nodal status. Using both imaging sequences, quantitative measurements including the largest lymph node dimension, cortical thickness and the ratio of cortical thickness to largest dimension were significantly higher in the node-positive group. In addition, cortical thickness proved to be the most discriminative quantitative measurement to predict positive lymph nodes using both sequences.

Axillary MR imaging are performed complementary to breast MR routinely, mostly for staging of breast cancer [19, 20]. A prior study reported that the role for MR is limited in the preoperative staging of axillary in breast cancer patients with poor sensitivity (43.0–50.0%), specificity (78.0–84.0%) and accuracy (68.0–72.0%) [21] and insisted only aggressive tumors or those with a later stage will more often show accurate MR findings. According to our results, standard MR and dedicated axillary MR imaging sequences demonstrated similar diagnostic performance to differentiate positive and negative axillary lymph nodes in both screening and diagnostic setting, with 64.7 and 66.2% sensitivity (*P* > 0.999), 94.0 and 93.3% specificity (*P* = 0.581), 94.3 and 94.4% NPV respectively (*P* = 0.715). Prior studies using dedicated axillary coil reported a median sensitivity of 84.7% (range, 73.0–94.6%) and NPV of 95.0% (range, 78.0–98.0%) [10, 22–24]. In contrary, using a coil covering both breast and axilla, lower median sensitivity of 82.0% (range, 33.3–97.0%) with NPV of 82.6% (range 1.9–95.7%) were observed [6, 7, 25–28]. Indeed, there have been many various attempts to evaluate axillary lymph nodes. A systemic review paper [5] concluded that using unenhanced T1 weighted or T2 weighted and USPIO enhanced T2*w sequences in combination with dedicated axillary protocol allows most acceptable sensitivity of 84.7% and NPV of 95.0%, allowing omission of SLNB during surgery; that dedicated axillary protocol is superior to standard protocol including both breast and axilla in the same field of view. Axillary MR imaging is often affected by respiratory movement artifacts from the thoracic wall and discrimination between lymph nodes and adjacent vasculatures can be challenging [15]. Though, with further technical refinement and improved resolution of breast MR imaging, the use of breast coil that covers the axillary region provide sufficient and good imaging quality of axilla. Our results demonstrate comparable sensitivity and NPV of standard MR imaging for axillary lymph node evaluation for both screening and diagnostic settings, that there is no added benefit of dedicated axillary MR sequence, which justify for omission of extra exam or MR scanning time.

Our results showed that cortical thickness is the most discriminative feature of suspicious lymph nodes with high AUC values, both using standard MR (0.823) and dedicated axillary MR sequences (0.846). Using both imaging sequences, largest lymph node dimension and the ratio of cortical thickness to largest dimension were also significantly higher in the positive lymph nodes. In addition, largest lymph node dimension, cortical thickness and the ratio of cortical thickness to largest dimension using both imaging sequences revealed strong agreement (all, > 0.70). Scaranelo et al. [6] reported that a cortical thickness greater than 3 mm was highly associated with malignancy (*P* < 0.001) and was the most reliable measurement to predict axillary metastasis, which is concordant to our finding. In a study evaluating axillary dedicated MR sequence, maximum cortical thickness, short and long axis length, relative T2 value, absence of fatty hilum (*P* < 0.001, respectively) and eccentric cortical thickening (*P* < 0.003) were found to be significantly different between positive and negative axillary lymph nodes. When four of the previous findings were noted, the specificity was about 90.0% [29]. Chung et al. also reported similar results showing that the mean size of the nodes in positive axillary lymph nodes is significantly larger [30].

With increasing incidence of breast cancer screening, abbreviated MR imaging has emerged which consists of pre-contrast and one early post-contrast T1 weighted series, post-contrast subtraction sequence and subtraction reconstructed imaging data for interpretation. This more simplified MR protocol decreases technologist and magnet time, decrease radiologist reading time and decrease overall cost, which makes breast MR a more viable screening option. Previous studies have shown similar cancer detection rates, PPV and/or NPV versus conventional MR imaging [17]. We found that standard MR imaging sequence demonstrate comparable high diagnostic performance to dedicated axillary MR sequence, suggesting standard MR imaging sequence alone is sufficient for lymph node evaluation and omission of dedicated axillary MR imaging sequence can be justified in abbreviated MR protocol.

This study has several limitations. First, this was a retrospective study and conducted in a single tertiary referral center with a large number of known cancer patients (72.7%). There may have been bias in the selection of patients for this study. Second, our statistical test was performed according to the number of patients affected axillae instead of the total number of involved lymph nodes. Thus, patients surgically treated with SLNB or ALND were unable to obtain a precise correlation of the visualized and/or interpreted lymph nodes. We assumed that the pathologic lymph node of the most suspicious node, assessed by imaging criteria, correlate with the overall diagnosis. Third, the primary purpose of this study was screening to identify whether the patient has any positive lymph nodes or not, not in setting of staging for breast cancer. Fourth, we excluded patients treated with neoadjuvant chemotherapy, which may have caused relatively low sensitivity. Lastly, breast MRI protocols were non-uniform (1.5 T and 3 T) during the study period, which may have affected diagnostic performance. Image analysis was performed in the same manner, regardless of the MRI systems.

## Conclusion

Our study revealed that the diagnostic performance of standard breast MR imaging sequence in the evaluation of axillary lymph node is comparable to dedicated axillary MR imaging sequence in women who underwent breast MR imaging. Using standard MR imaging sequence, we reliably classified node-positive versus node negative patients. Our results demonstrate the justification of omitting of dedicated axillary imaging sequence, especially in the abbreviated breast MR for breast cancer screening. Further studies should be performed to evaluate the value of our results with respect to guiding patient management regarding axillary lymph nodes.

## Supplementary information

**Additional file 1.**

## Data Availability

The datasets analyzed during the current study are available from the corresponding author on reasonable request.

## Abbreviations

MR: Magnetic resonance
ALND: Axillary lymph node dissection
LD: Largest dimension
CT: Cortical thickness
