# Supplementary material for: Diagnostic performance of standard breast MR imaging compared to dedicated axillary MR imaging in the evaluation of axillary lymph node
Source: BMC Med Imaging. 2020 May 1;20:45. doi: 10.1186/s12880-020-00449-4 (PMC7195753; doi:10.1186/s12880-020-00449-4)
Supplement: Supplementary file 1 — Additional file 1. [file 12880_2020_449_MOESM1_ESM.docx]

Supplementary Table 1. Breast MRI Acquisition Protocols

| Standard MRI Protocol | 1.5 T | 3 T |
| --- | --- | --- |
| Scan dimension | Bilateral Axial | Bilateral Axial |
| T2 Weighted Imaging | | |
| TR/TE (msec) | 6700/74 | 1100/131 |
| Field of view (mm) | 300 x 300 | 341 x 210 |
| Thickness (mm) | 1.5 | 1.5 |
| Matrix | 448 x 448 | 256 x 416 |
| Dynamic T1 Weighted Imaging | | |
| TR/TE (msec) | 5.2/2.4 | 5.6/2.5 |
| Thickness (mm) | 0.9 | 0.9 |
| Matrix | 384 x 384 | 360 x 360 |
| T1 Weighted Dedicated Axillary MRI Protocol | | |
| TR/TE (msec) | 5.6/2.6 | 4.1/1.3 |
| Flip angle | 10° | 12° |
| Thickness (mm) | 1.5 | 1.0 |
| Matrix | 298 × 352 | 340 × 380 |

TR=repetition time, TE=Echo time
